# Collecting and preserving bark and ambrosia beetles (Coleoptera: Curculionidae: Scolytinae & Platypodinae)

**DOI:** 10.1371/journal.pone.0265910

**Published:** 2022-04-15

**Authors:** Jiri Hulcr, Demian F. Gomez, Andrew J. Johnson

**Affiliations:** 1 School of Forest, Fisheries and Geomatics Sciences, University of Florida, Gainesville, Florida, United States of America; 2 Texas A&M Forest Service, Austin, Texas, United States of America; Institute of Systematics and Evolution of Animals Polish Academy of Sciences, POLAND

## Abstract

This protocol describes the different methods to collect and preserve bark and ambrosia beetles, detailing collecting tools, recording relevant data, and optimizing step-by-step methods to extract beetles from twigs, branches, bark, and trunks. It elaborates on trapping techniques, tools, lures, baits, and beetle preservation. The main rule of manual collecting is to not attempt to pry the insect out of the wood or bark, but instead, remove the wood/bark away from the beetle: gently and systematically. The main rule of trapping is that there is no general attractant; instead, attractants and traps should reflect the ecology of the targeted beetle taxa.

## Introduction

Insect sampling and insect collections are some of the most important components of entomological research and teaching. However, certain taxa, particularly small wood borers, are challenging to sample. Bark and ambrosia beetles (Coleoptera: Curculionidae: Scolytinae and Platypodinae) are some of the smallest and most common insects in natural, urban, and commercial forests. While the vast majority breed in dead or dying tissues and are harmless, some species have caused devastating damage across within both native and introduced range. Just in the last decades, more than 300 million redbay trees have been killed by laurel wilt [1], millions of ha of pine trees have been killed by the mountain pine beetle [2], and the *Euwallacea fornicatus* species complex has caused significant impacts to orchards and natural forests around the world [3–5].

Bark beetle sampling is an essential part of integrated management programs, including beetle surveillance and monitoring by government agencies. For example, the Cooperative Agricultural Pest Survey (CAPS) by the USDA APHIS, which conducts national and state surveys, together with the Forest Service’s Early Detection and Rapid Response program (EDRR), are responsible for post-introduction detection of pests [6]. However, these efforts focus on traps and a few selected lures, leaving the majority of the bark and ambrosia beetle diversity unsampled.

Contemporary biobanks are increasingly focused on collecting and storing the specimen with its context [7]. In the case of wood borers, this may include a sample of the hosts tree, the associated fungi, or the microbiome. In bark and ambrosia beetles, a special emphasis should be placed on sampling the fungal symbionts, given the major economic and ecological significance of some of them [1, 8–11]. The symbiotic fungi that these beetles carry have become the focus of a renewed research interest in the recent decade [12].

Bark and ambrosia beetle species are distinguished by small and subtle morphological differences [13–15]. Therefore, to reliably identify species, quality samples must retain all morphological structures. This requires dexterity and a specialized sequence of steps in retrieving the specimens from wood.

Also of increasing importance has been the collecting of high quality samples for DNA studies. Advancements in molecular biology techniques have benefited numerous research fields related to forest health, including phylogenetics and systematics, invasion ecology, and forest pest diagnostics [16, 17]. Molecular identification is now the standard when studying the symbiotic relationships between the beetles and vectored fungi [12]. Despite some limitations of the DNA barcoding approach, some molecular markers have shown to be effective for identification and delimitation of scolytines, particularly when coupled with morphological evidence in a phylogenetic/systematic framework [14, 18].

Despite extensive treatment of wood boring insects in literature on collecting and preserving insects, little is mentioned regarding manual extraction from wood samples [19]. Successful collection of bark and ambrosia beetle needs to be guided by the targeted beetle species, with different tools and trapping systems needed depending on feeding ecology (i.e., phloeophagous vs. xylomycetophagous species), beetle size, and chemical ecology. Box cutters, hand saws, chisels, and pruning clippers, are used for different parts of the tree, depending on where the target beetle occurs: twigs, branches, trunk, or bark. The main principle we recommend for extracting high-quality specimen of bark and ambrosia beetles is to not try to remove the beetle from the wood or bark; instead, to remove the wood/bark away from the beetle: carefully and systematically. In terms of lure choice, we suggest that, despite of the many attempts to use a “generic” lure for all bark and ambrosia beetles (such as ethanol), no such lure has been devised yet. Instead, each lure attracts species whose ecology it reflects [20–22].

Here we present a protocol to collect bark and ambrosia beetles, with step-by-step guidelines to obtain high quality samples. It describes the different methods to collect and preserve bark ambrosia beetles, detailing collecting tools, relevant data, and optimized methods to extract beetles from twigs, branches, bark, and trunks. Moreover, it elaborates on trapping techniques, tools, lures, baits, and beetle preservation.

This protocol is part of a repository hosted on Protocols.io, as part of the public workspace ‘Bark Beetle Mycobiome (BBM) research coordination network’ (https://www.protocols.io/workspaces/protocols-bark-beetle-mycobiome). Bark Beetle Mycobiome is a global research community reinvigorating the science of bark beetle-fungus symbiosis [12].

## Materials and methods

The protocol described in this peer-reviewed article is published on protocols.io, dx.doi.org/10.17504/protocols.io.bpjdmki6 and is included for printing S1 File with this article. This publication provides context for the Protocol. For the actual beetle sampling, the Protocol should be followed.

## Expected results

Although studies with bark and ambrosia beetles have been increasing in the last decades, few resources provide detailed methods to collect high quality samples. Our method based, based on taking the wood away from the beetle and not the beetle out of wood, will provide collectors with the methodology needed to collect wood borers.

The collecting methods proposed here have shown to be highly effective for several studies across the world, including pre-invasion assessments of potential invasive threats [23, 24], biodiversity studies [25, 26], and citizen science projects.

## Supporting information

S1 File The protocol described in this peer-reviewed article is published on protocols.io, dx.doi.org/10.17504/protocols.io.bpjdmki6. The protocol is also available as a Supplementary Information for this publication.
(PDF)
