# Supplementary material for: Collecting and preserving bark and ambrosia beetles (Coleoptera: Curculionidae: Scolytinae & Platypodinae)
Source: PLoS One. 2022 Apr 15;17(4):e0265910. doi: 10.1371/journal.pone.0265910 (PMC9012363; doi:10.1371/journal.pone.0265910)
Supplement: S1 File — The protocol is also available as a Supplementary Information for this publication. (PDF) [file pone.0265910.s001.pdf]

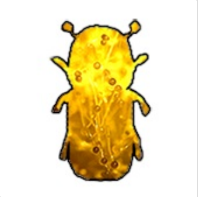

2

Nov 09, 2020

Collecting Bark and Ambrosia Beetles V.2

1

Jiri Hulcr[1], Andrew J. Johnson[1], Demian F Gomez[1]

[1]University of Florida

2

dx.doi.org/10.17504/protocols.io.bpjdmki6

Protocols Bark Beetle Mycobiome

Bark Beetle Mycobiome Research Coordination Network

This protocol describes the different methods to collect and preserve bark ambrosia beetles.

This protocol is part of the Bark Beetle Mycobiome (BBM) Research Coordination Network. For more information on the BBM international network: Hulcr J, Barnes I, De Beer ZW, Duong TA, Gazis R, Johnson AJ, Jusino MA, Kasson MT, Li Y, Lynch S, Mayers C, Musvuugwa T, Roets F, Seltmann KC, Six D, Vanderpool D, & Villari C. 2020. Bark beetle mycobiome: collaboratively defined research priorities on a widespread insect-fungus symbiosis. Symbiosis 81: 101–113 https://doi.org/10.1007/s13199-020-00686-9.

DOI

dx.doi.org/10.17504/protocols.io.bpjdmki6

Jiri Hulcr, Andrew J. Johnson, Demian F Gomez 2020. Collecting Bark and Ambrosia Beetles. **protocols.io**
https://dx.doi.org/10.17504/protocols.io.bpjdmki6
Bark Beetle Mycobiome Research Coordination Network

2020 Featured Protocols

document ,

Nov 09, 2020

Nov 09, 2020

Citation: Jiri Hulcr, Andrew J. Johnson, Demian F Gomez Collecting Bark and Ambrosia Beetles
https://dx.doi.org/10.17504/protocols.io.bpjdmki6

This is an open access protocol distributed under the terms of the **Creative Commons Attribution License** (https://creativecommons.org/licenses/by/4.0/), which permits unrestricted use, distribution, and reproduction in any medium,

44357

Part of collection

2020 Featured Protocols

This protocol describes the different methods to collect and preserve bark ambrosia beetles.

This protocol is part of the Bark Beetle Mycobiome (BBM) Research Coordination Network. For more information on the BBM international network: Hulcr J, Barnes I, De Beer ZW, Duong TA, Gazis R, Johnson AJ, Jusino MA, Kasson MT, Li Y, Lynch S, Mayers C, Musvuugwa T, Roets F, Seltmann KC, Six D, Vanderpool D, & Villari C. 2020. Bark beetle mycobiome: collaboratively defined research priorities on a widespread insect-fungus symbiosis. Symbiosis 81: 101–113 https://doi.org/10.1007/s13199-020-00686-9.

The selection of tools partly depends on which types of scolytine beetles you are mostly interested in. For example, for twig bark beetles you will not need any of the heavy duty hardware. On the other hand, trying to pry ambrosia beetles out of a branch with a knife routinely leads to squashed specimens (for xylophages we recommend sawing out a wood "cookie" with the gallery in it, splitting it out with a chisel, and peeling pieces off with clippers, until you get at the beetle). Our recommendations of brands are based on years of experience, not on any relationship with the vendors.

The rule number one: do not try to take the beetle out of wood/bark, instead, take the wood/bark away of the beetle: slowly, gently, systematically. Also, do not break twigs by hand and do not use a knife.

Collecting tools

The selection of tools partly depends on which types of scolytine beetles you are mostly interested in. For example, for twig bark beetles you will not need any of the heavy duty hardware.

Many researchers take a knife as a first tool of choice. A knife tends to be a poor bark beetling tool that results in many squashed specimens. Our recommendations of brands are based on years of experience, not on any relationship with the vendors.

Essential

1. box cutter (heavy duty)
2. anvil-style clippers (we use Bahco anvil pruner)
3. hatchet (we use Kershaw Camp Axe)
4. folding saw
5. broad wood chisel
6. vials/cryo-tubes with ethanol (screw-top; never use snap-top vials)
7. pre-cut labels
8. label pen (we use 0.5 Pigma MICRON archival pen) or pencil
9. soft forceps on a string, so you can hang it on your neck, otherwise you lose at at the first collecting

Citation: Jiri Hulcr, Andrew J. Johnson, Demian F Gomez Collecting Bark and Ambrosia Beetles https://dx.doi.org/10.17504/protocols.io.bpjdmki6

This is an open access protocol distributed under the terms of the **Creative Commons Attribution License** (https://creativecommons.org/licenses/by/4.0/), which permits unrestricted use, distribution, and reproduction in any medium,

site
10. tool bag
11. bullet box for vials
12. magnifying glass
13. Collection bags, various sizes (for carrying sticks to dissect later)
14. Any tubes or other container with holes poked (for live specimens)
15. Kimwipes/tissues (for live specimens)

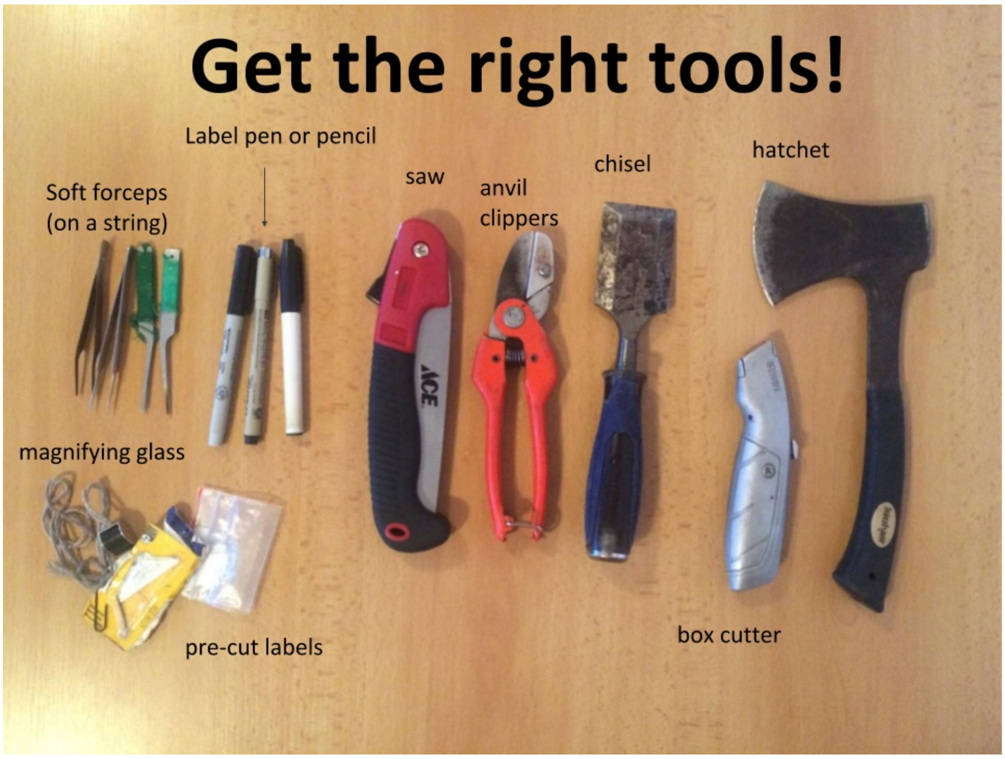

Not essential but useful
1. Cell phone macro lens for photos in situ, and for georeferenced records
2. Cell phone with iNaturalist for identifying host plants
3. Folding saw for larger logs, our preferred is Agawa Canyon. In most cases, it replaces the need for a chain saw.
4. scalpel (for very small galleries)
5. if sampling living beetles: Vials with sawdust media - each will need a hole pricked in the top after beetle is inserted. See separate recipe for beetle-vial media.
6. collecting notebook/log
7. aspirator (pooter)

Collection data

These may be used for classifying the wood substrate the beetles live in.

levels of humidity
1. in water
2. on ground-buried
3. on ground

Citation: Jiri Hulcr, Andrew J. Johnson, Demian F Gomez Collecting Bark and Ambrosia Beetles
https://dx.doi.org/10.17504/protocols.io.bpjdmki6

This is an open access protocol distributed under the terms of the **Creative Commons Attribution License** (https://creativecommons.org/licenses/by/4.0/), which permits unrestricted use, distribution, and reproduction in any medium,

4. above ground- moist
5. above ground-dry

levels of decay

1. fresh, sap present
2. freshly dead, bark easy to peel, sap absent
3. fungi present, bark loose
4. bark falls off; other insects
5. rotten, past main colonization

Collecting perfect specimens:

For Twig Beetles: peel the bark with a box cutter and gently remove the beetles with soft forceps.

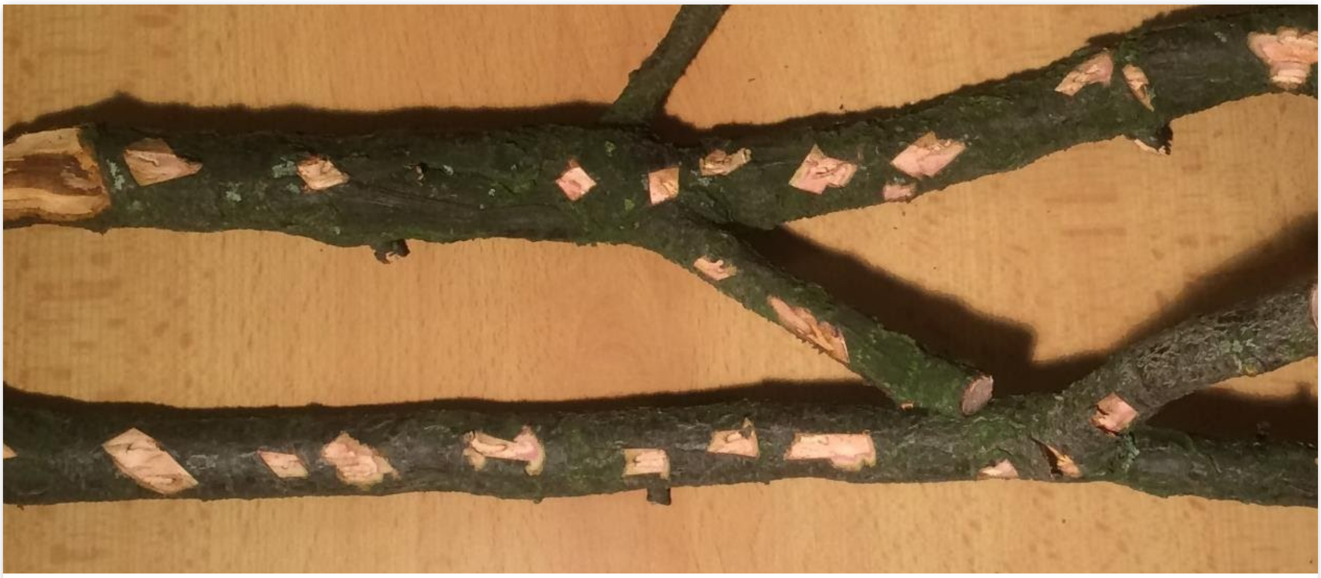

For Beetles in branches (for example ambrosia beetles):

1. Cut off small piece of branch with a saw
2. Clip wood with clippers
3. Remove the beetle

Citation: Jiri Hulcr, Andrew J. Johnson, Demian F Gomez Collecting Bark and Ambrosia Beetles https://dx.doi.org/10.17504/protocols.io.bpjdmki6

This is an open access protocol distributed under the terms of the **Creative Commons Attribution License** (https://creativecommons.org/licenses/by/4.0/), which permits unrestricted use, distribution, and reproduction in any medium,

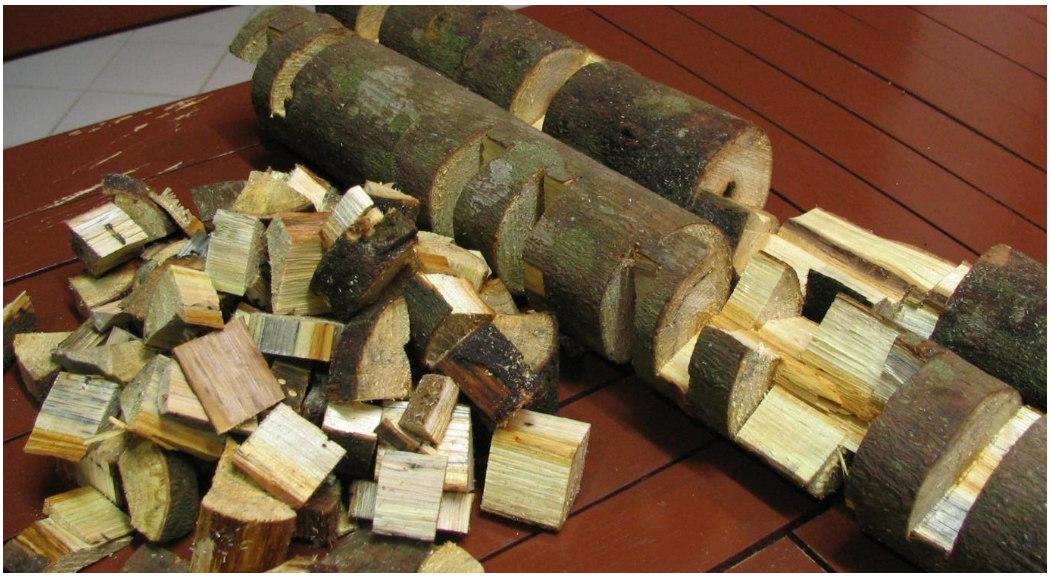

For Beetles under Bark: Peel off large section of the bark and remove beetles with soft forceps.

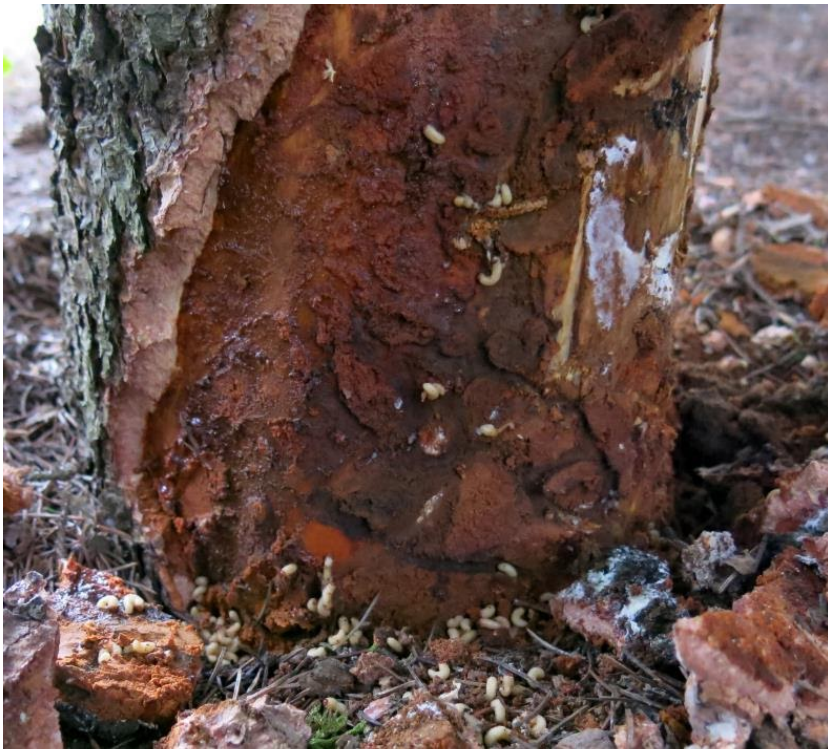

For beetles in a tree trunk:

1. Cut off a “cookie” or a wedge from the trunk
2. Cut pieces off with a chisel
3. Clip wood with clippers

Citation: Jiri Hulcr, Andrew J. Johnson, Demian F Gomez Collecting Bark and Ambrosia Beetles
https://dx.doi.org/10.17504/protocols.io.bpjdmki6

This is an open access protocol distributed under the terms of the **Creative Commons Attribution License** (https://creativecommons.org/licenses/by/4.0/), which permits unrestricted use, distribution, and reproduction in any medium,

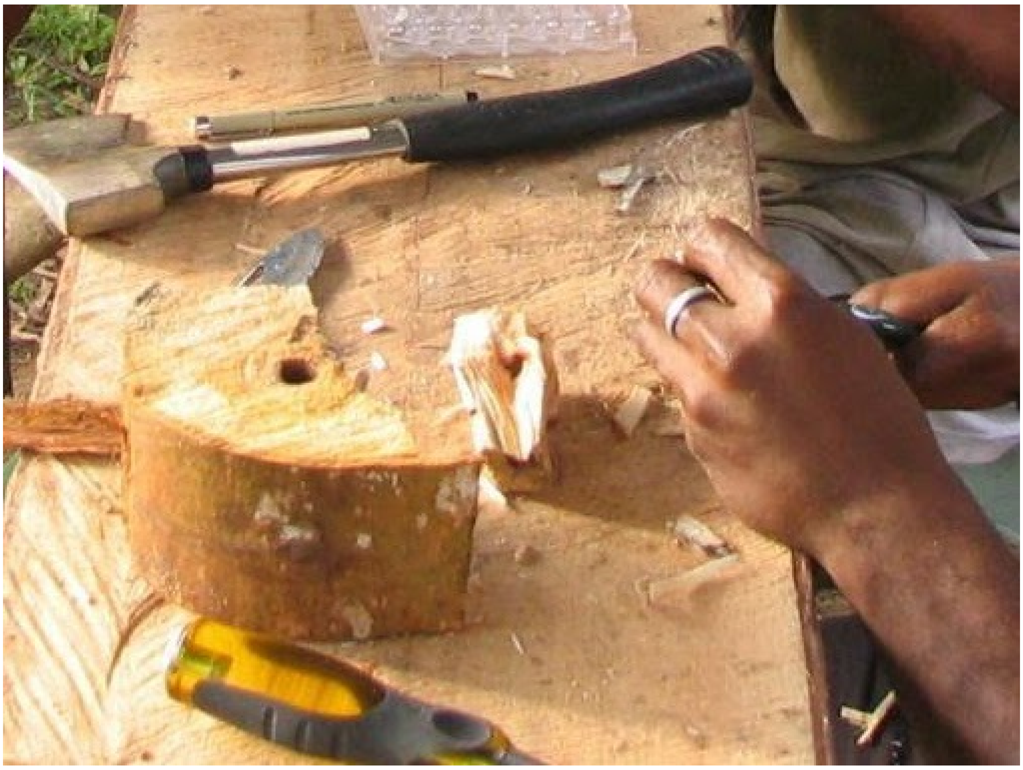

Trapping

Funnel/bottle trapping

1. Lindgren traps or bottle traps (2L bottles or materials to make them (see below, also: http://ambrosiasymbiosis.org/wp-content/uploads/2013/03/bbtrap.pdf)
2. Knife for constructing bottle traps, cutting rope
3. Rope and/or twine
4. Twist-ties or cable ties
5. Tape (electrical)
6. Lures, or >95% un-denatured etoh
7. Bags for lures/ethanol
8. Squirt-bottle for re-filling etoh bags and tubes
9. Plastic pasteur pipette
10. Collection bags, various sizes (for carrying sticks to dissect later)
11. For live specimens, vials with wood flour media
12. Labeling equipment (Pigma pens, labels)

Lures

For ethanol lure – attach a mini ziploc bag made of thin plastic, fill half way with ethanol, and poke many hole above the ethanol level.
Take ethanol squirt bottle with you to the field and fill them on site. Change every couple of days (otherwise ethanol will soak up water from the environment).

For dead and well preserved beetles: use upside-down bottle with ethanol in the bottom (no need for ethanol lure bag).

Citation: Jiri Hulcr, Andrew J. Johnson, Demian F Gomez Collecting Bark and Ambrosia Beetles
https://dx.doi.org/10.17504/protocols.io.bpjdmki6

This is an open access protocol distributed under the terms of the **Creative Commons Attribution License** (https://creativecommons.org/licenses/by/4.0/), which permits unrestricted use, distribution, and reproduction in any medium,

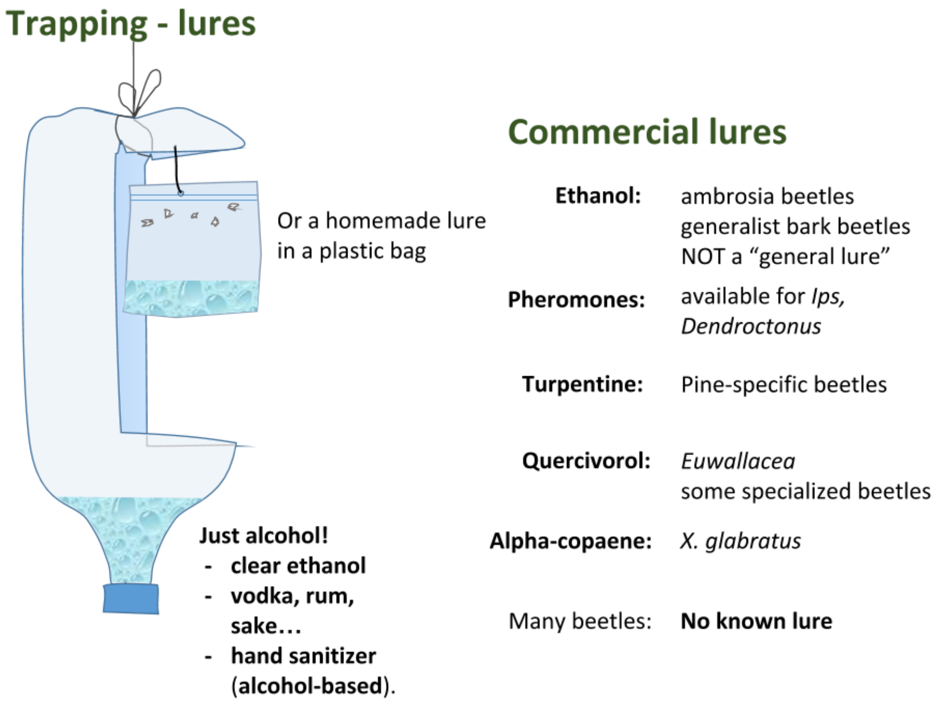

For live beetles: use smaller bottle attached to the bottom of flight-intercept bottle. The connector can be made of many things, for example plumbing insulation foam or electric tape. Cover the bottom of the small bottle with shreds of paper towel. Big chunks of paper are not good – then don't cover the slippery bottom, and beetles have nothing to grab on. Remove live beetles every couple of days (daily is best), and change towel shreds every two weeks at least.

Light-trapping

1. Light source of choice, batteries, etc.
2. We typically use at least two white (UltraFire Sk98 Cree Xml-t6) and two black (UltraFire SK98 UV) flashlights per sheet
3. Voltage converter if necessary
4. glasses with UV protection
5. White sheet (Queen size is best)
6. Rope/twine to hang sheet
7. Clips/clothespins
8. Bowl/tray for ethanol

Not essential but useful

1. Ethanol-resistant labeling markers
2. Tarp for cutting-up wood indoors (easy clean-up)
3. Multi-tool
4. Collecting notebook/log
5. Aspirator (pooter)
6. Electric or regular chain saw (remove oil if packing, also pack oil and allen wrench)
7. Scalpel (for very small galleries)
8. Pin/teasing needle (small galleries, can also just use hard forceps)

Citation: Jiri Hulcr, Andrew J. Johnson, Demian F Gomez Collecting Bark and Ambrosia Beetles https://dx.doi.org/10.17504/protocols.io.bpjdmki6

This is an open access protocol distributed under the terms of the **Creative Commons Attribution License** (https://creativecommons.org/licenses/by/4.0/), which permits unrestricted use, distribution, and reproduction in any medium,

9. Watch glasses for IDing in the lab, under a scope.

Baits

Bait branches

A bait branch bundle is an amazing way to collect twig borers. These are some of the most diverse bark and ambrosia beetles. Make sure you use a meaningful host, and do it in the correct season. Ideally you would want to pull it up to the canopy, or at least get it off the ground, but not in direct sunlight. The bigger bundle the better! The one on this picture is pretty small.

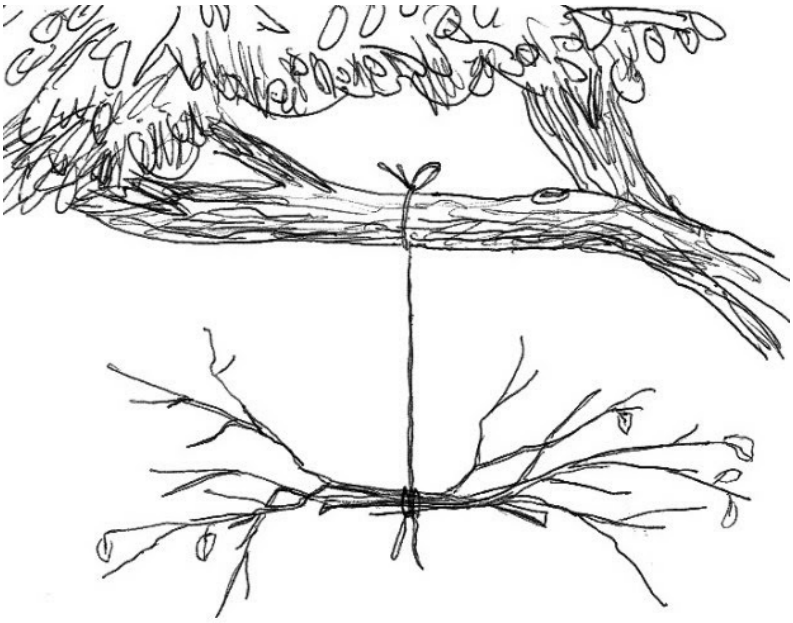

Storing Beetles

Citation: Jiri Hulcr, Andrew J. Johnson, Demian F Gomez Collecting Bark and Ambrosia Beetles https://dx.doi.org/10.17504/protocols.io.bpjdmki6

This is an open access protocol distributed under the terms of the **Creative Commons Attribution License** (https://creativecommons.org/licenses/by/4.0/), which permits unrestricted use, distribution, and reproduction in any medium,

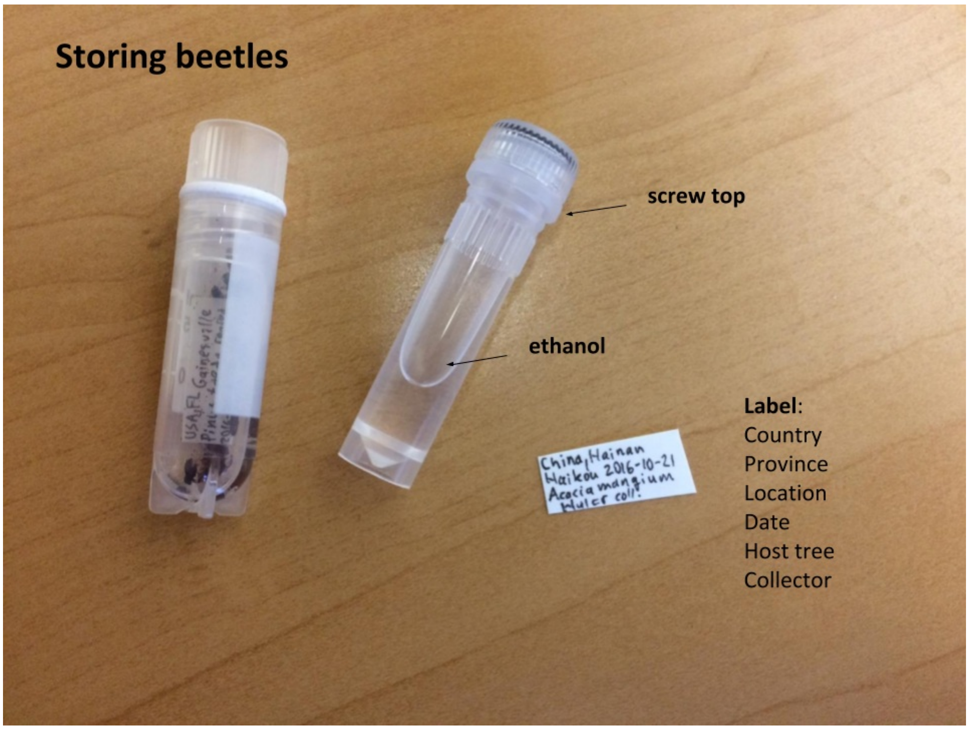

Beetle Identification after collecting.

Larvae can be collected if DNA identification will be carried out. If it is not needed, well preserved specimens will suffice. A skilled taxonomist can identify species faster and better than molecular tools in most cases.

Reporting Bark Beetles

1 - Take as many photographs as possible of the damage, galleries, symptoms on the tree, etc.

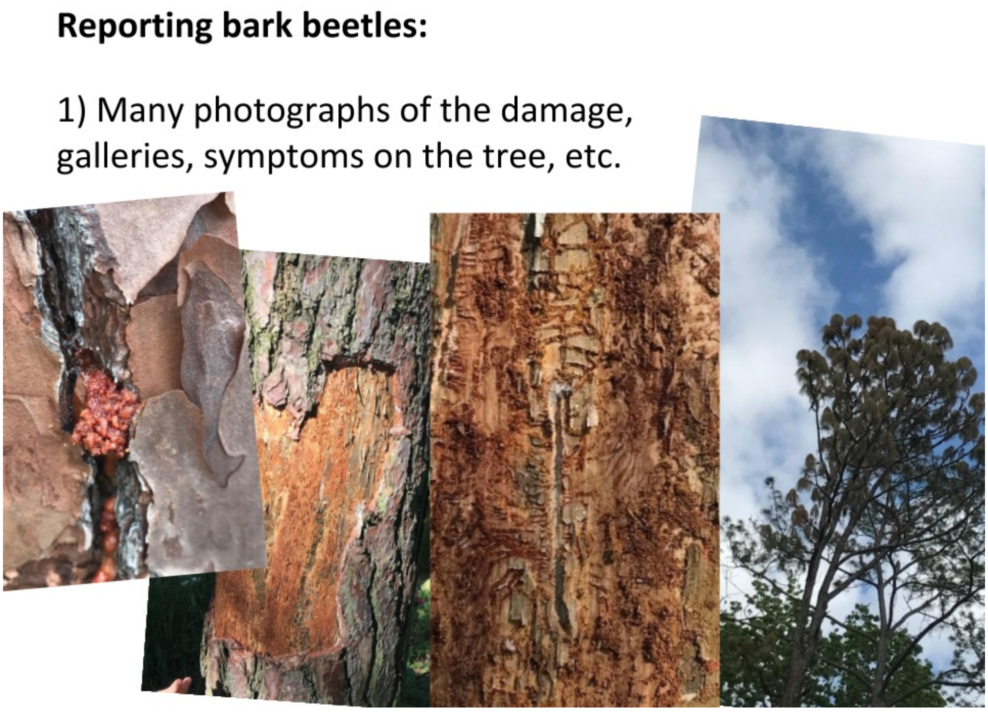

2 - Photograph the beetles:

Citation: Jiri Hulcr, Andrew J. Johnson, Demian F Gomez Collecting Bark and Ambrosia Beetles
https://dx.doi.org/10.17504/protocols.io.bpjdmki6

This is an open access protocol distributed under the terms of the **Creative Commons Attribution License** (https://creativecommons.org/licenses/by/4.0/), which permits unrestricted use, distribution, and reproduction in any medium,

- Dorsal shape
- Lateral shape
- Declivity
- Antennae

Mobile phone lenses are usually sufficient.

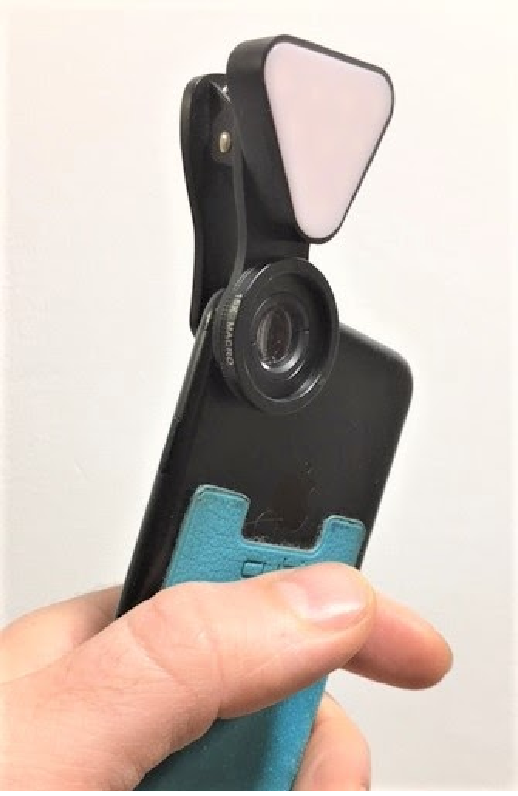

3 - Collect and ship the beetle

- Screw-top sealed plastic vial
- A few drops of ethanol
- Cotton and label inside
- Padded envelope

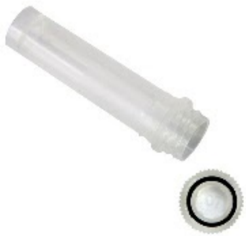

For international travel

Arrange for in advance:

- Permits for shipping fungi arranged
- Vaccinations if required/recommended for destination
- International calling setup on cell phone
- International driver’s license (if renting car)
- Pcard/personal credit/debit card travel authorizations

Citation: Jiri Hulcr, Andrew J. Johnson, Demian F Gomez Collecting Bark and Ambrosia Beetles
https://dx.doi.org/10.17504/protocols.io.bpjdmki6

This is an open access protocol distributed under the terms of the **Creative Commons Attribution License** (https://creativecommons.org/licenses/by/4.0/), which permits unrestricted use, distribution, and reproduction in any medium,

- Accommodation reservations
- *For University of Florida*: travel authorization submitted, completed online travel registration checklist (http://ufic.ufl.edu/travelregistration.html), and team Assist insurance set up (card printed to take on trip).

Things to make sure are available at destination/hosting lab in advance (if needed)

- Plates plus potentially other lab tools, consumables listed above (allow time for hosting lab to order to ensure arrival in time)
- Autoclave
- Glassware for autoclaving media
- Sterile hood
- Bulk ethanol (lots necessary if trapping – illegal to fly with large amounts of ethanol)
- Microscopes
- Microscope camera
- Printing capabilities
- WiFi and printing capability
- Incubators
- traps to borrow

Citation: Jiri Hulcr, Andrew J. Johnson, Demian F Gomez Collecting Bark and Ambrosia Beetles
https://dx.doi.org/10.17504/protocols.io.bpjdmki6

This is an open access protocol distributed under the terms of the **Creative Commons Attribution License** (https://creativecommons.org/licenses/by/4.0/), which permits unrestricted use, distribution, and reproduction in any medium,
